# Knowledge and Attitude of General Pakistani Population Towards Antibiotic Resistance

**DOI:** 10.7759/cureus.4266

**Published:** 2019-03-18

**Authors:** Ramsha Akhund, Fatima Jamshed, Hassam A Jaffry, Hamza Hanif, Sundus Fareed

**Affiliations:** 1 General Medicine, Jinnah Sindh Medical University, Karachi, PAK; 2 Pediatrics, Jinnah Sindh Medical University, Karachi, PAK; 3 Internal Medicine, Jinnah Postgraduate Medical Center, Karachi, PAK; 4 Internal Medicine, Jinnah Sindh Medical University, Karachi, PAK; 5 Internal Medicine, Civil Hospital, Karachi, PAK

**Keywords:** knowledge and attitude, antibiotics and antibiotic resistance, antibiotic stewardship, antibiotic prescribing, self-prescription

## Abstract

Introduction

The emergence and continuous spread of drug resistant bacteria has become one of the leading health concerns globally. Persistent failure to develop and/or discover new antibiotics along with irrational use of existing antibiotics is associated with rise in antibiotic resistance. There is poor understanding of antibiotics usage and their preciousness among the masses which result in careless utilization and hence, the emerging antibiotic resistance. The aim of this study is to evaluate the knowledge and attitude of Pakistanis towards antimicrobial resistance (AMR).

Methods

This observational cross-sectional survey was designed in the form of an online pro forma circulated in January 2019. It was a self-structured pro forma which included age, gender, and 10 questions - five to assess the knowledge and five to assess the attitude towards AMR. Each question was to be responded with a “Yes” or a “No.” For knowledge assessing questions “do not know” was also an option. Data was entered and analysed using SPSS version 22 (IBM Corp., Armonk, NY).

Results

Of 1,132 participants, 837 (73.9%) thought that it was alright to stop antibiotics course whenever they felt better, 505 (44.6%) thought that frequent and unnecessary use of antibiotics actually decrease their effectiveness, and 208 (18.4%) participants thought it was correct to take antibiotics for cold and influenza. There were 157 (13.9%) participants who had not followed the duration of treatment as given in their doctor’s prescription, 49 (4.4%) who had changed their antibiotic dose without doctor consultation, 467 (41.3%) who had reused leftover antibiotics from their previous prescription, 700 (61.8%) who had suggested it to their doctors to prescribe them antibiotics and 378 (33.4%) participants who had purchased antibiotics without any prescription in the last one year.

Conclusion

Pakistani individuals are not as knowledgeable about antibiotic resistance as severe the issue is in this region. Their attitude towards utilization of antibiotics is not very promising. It becomes essential to initiate antibiotic stewardship programs and educate the masses regarding efficacious and safe use of antibiotics in this region.

## Introduction

The emergence and continuous spread of drug resistant bacteria has become one of the leading health concerns not only for humans, but also for animals, plants, and crops; hence the overall environmental health [1]. Antibiotics treatment is one of the main approaches of modern medicine to combat infections. The early 19^th^ century was named "Golden Era" due to discovery of many new antibiotics [2]. However, the golden period did not last for long as the researchers were unable to keep up with the rapidly emerging resistant pathogens. Persistent failure to develop and/or discover new antibiotics along with irrational use of existing antibiotics is predisposing to the emergence of antimicrobial resistance (AMR) [3].

Another major reason for antibiotic resistance is overuse and misuse. Although, medical literature has always strongly advised cautious use of antibiotics, they still remain highly over-prescribed all across the globe. Evidence from various studies has stated that treatment indications, agent choice, and antibiotic therapy duration were not medically correct in 30%-50% of the cases [4, 5]. Irrational antibiotics use does not only indicate lack of patient compliance towards physicians’ instructions, but inappropriate antibiotic prescription from the physicians is also not uncommon. Correct indication, right drug and dosage, drug of first choice, appropriate period of use, and lower treatment cost, all contribute to rational and judicious antibiotic prescription [6].

Patients and the general population contribute to another important factor leading to antibiotic resistance - self-medication [7]. Around 2/3^rd^ of all oral antibiotics used globally are taken without prescriptions and are used for incorrect indications such as tuberculosis, malaria, and for childhood viral infections [8].

In a study conducted with physicians, it was seen that 20% of their antibiotic prescriptions were influenced by their patients' choice and demand [9]. This is reinforced by another study where 40% individuals believed that antibiotics should be prescribed for all flu-like symptoms and 30-50% did not understand the difference between antibiotics, antipyretics, and anti-inflammatory drugs [10]. These findings indicate poor understanding of antibiotics usage and their preciousness among the masses which result in careless utilization and hence, the emerging antibiotic resistance. The aim of this study is to evaluate the knowledge and attitude of Pakistanis towards antibiotics resistance.

## Materials and methods

This observational cross-sectional survey was designed in the form of an online pro forma circulated via social media in January 2019. It was a self-structured pro forma which included age, gender, and 10 questions - five to assess knowledge and five to assess attitude. Each question was to be responded with a “Yes” or a “No.” For knowledge assessing questions “do not know” was also an option. Since it was a self-structured pro forma, its internal reliability was first calculated through a pilot survey (n = 20). Data was entered and analysed using SPSS version 22 (IBM Corp., Armonk, NY). Internal reliability deduced via Cronbach alpha was 0.79. Frequency and percentages were calculated for categorical variables including questions for assessing knowledge and attitude. Mean and standard deviation (SD) were calculated for continuous variable of age.

## Results

One thousand one hundred and thirty-two pro formas were completed. There were 612 (54.06%) men and 520 (45.94%) women. The mean age of all participants was 35.84 ± 11.09 years.

**Figure 1:**
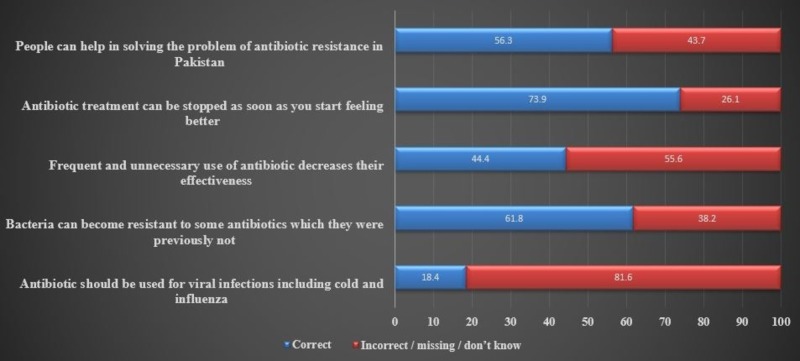
Assessment of knowledge about antibiotic resistance (%).

The responses to knowledge about antibiotics resistance are shown in Figure 1. It shows that 637 (56.3%) people knew that they could contribute to decrease antimicrobial resistance, 837 (73.9%) participants thought that it was alright to stop antibiotic course whenever they felt better, 505 (44.6%) individuals thought that frequent and unnecessary use of antibiotics actually decrease their effectiveness, 700 (61.8%) thought that bacteria could become resistant to antibiotics over time, and there were 208 (18.4%) participants who thought it was correct to take antibiotics for cold and influenza (Figure 1).

**Figure 2:**
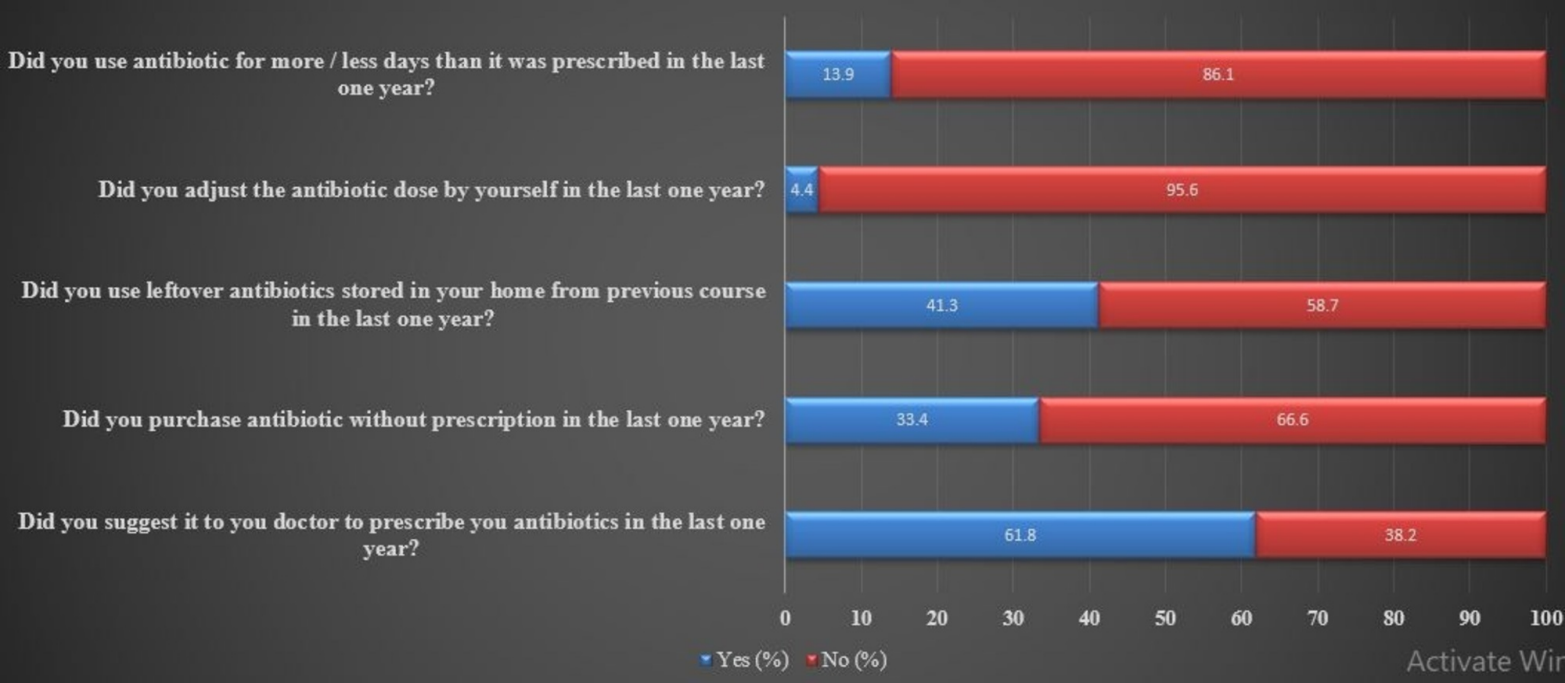
Assessment of attitude about antibiotic resistance (%).

The attitude of the participants towards antibiotics resistance was assessed by five questions as shown in Figure 2. It was seen that 157 (13.9%) participants had not followed the duration of treatment as given in their doctor’s prescription, however, only 49 (4.4%) changed their antibiotic dose without doctor consultation. There were 467 (41.3%) participants who used leftover antibiotics from their previous prescription. It was alarming that there were 700 (61.8%) participants who suggested it to their doctors to prescribe them antibiotics and 378 (33.4%) participants who had purchased antibiotics without any prescription in the last one year (Figure 2).

## Discussion

Irresponsible and inappropriate prescriptions of antibiotics are among the major reasons contributing to the rising antimicrobial resistance. Almost one-third of the participants in this study took antibiotics without prescription. A study published in 2016 stated that over the counter sales of antibiotics is one of the major reasons for higher prevalence of resistance in the developing countries [11]. Inappropriate antimicrobial use by patients even with true bacterial infections can lead to failure of treatment and can mask various clinical symptoms associated with the disease [12]. Incorrectly managed patients - either who received antibiotics in absence of bacterial infection or who received incorrect drug or who received treatment for sub-optimal duration - essentially took antibiotics without any benefit and efficacy. Non-prescription antimicrobial use has been linked with severe adverse events including death [13, 14]. In this study, 41% of the participants used left over antibiotics from their own or someone else’s previous antibiotic course. This practice of using left over antibiotics from previous antibiotic course may lead to increased use of antibiotics for short duration especially for viral infections, which may lead to antibiotic resistance [15]. There were 19% participants who believed that antibiotics can be taken in viral infections. A study published in 2017 stated that 18.3% of office-based visits for viral diseases resulted in antibiotic prescriptions [16].

The public health sector in Pakistan has recognized antibiotic resistance as its major health concern. In 2017, National Antimicrobial Resistance Action Plan was created for Pakistan. Its major strategic priority is “Development and implementation of a national awareness raising and behavioral change strategy on antimicrobial resistance [17].” This study serves as a supporter to this national action plan and highlights the key lacking in awareness and behaviors.

The urgent need for implementing antibiotic stewardship programs in Pakistan has been highlighted previously [18]. However, no aggressive measures have been taken yet. The attitude of the general population towards utilization of antibiotics is not very promising and needs immediate correction. Specialists of public health and infectious diseases in Pakistan have to join hands in initiating awareness programs and campaigns to educate the general masses regarding the preciousness of antibiotics.

## Conclusions

Pakistani individuals are not as knowledgeable about antibiotic resistance as severe the issue is in this region. Their attitude towards utilization of antibiotics is not very promising. The rapidly rising trend in antibiotic resistance is becoming a public health concern in Pakistan. It has become essential to initiate antibiotic stewardship programs and educate the masses regarding efficacious and safe use of antibiotics in this region.
